# Precocious Maturity

**Published:** 1916-04

**Authors:** 


**Precocious Maturity.** F. Beekman, Arch. of Paed., 1915, xxxii, 4, reports a case in a girl of 6½ years, mentally on the actual age, physically appearing as a girl of 16, with menstruation, mammary development, etc. Radiograms also showed corresponding ossification. There is a review of the literature.

